# Incidental discovery of abdominal splenosis mimicking metastatic disease during elective incisional hernia repair: a case report

**DOI:** 10.1186/s13037-026-00487-x

**Published:** 2026-05-21

**Authors:** Soniya Shankar Kalpana, Balaji Durairaj

**Affiliations:** Department of General Surgery, SRM Medical College and Hospital & Research Centre, Faculty of Medicine and Health Sciences, SRM IST, Kattankulathur, Chengalpattu [Dt], Tamil Nadu India

**Keywords:** Abdominal splenosis, Incisional hernia, Peritoneal metastasis mimic, Intraoperative decision making, Patient safety

## Abstract

**Background:**

Abdominal splenosis is a benign condition that occurs when splenic tissue is implanted within the abdominal cavity after splenic trauma or splenectomy. Most patients remain asymptomatic, and the condition is often detected incidentally. However, when multiple nodules are present, they can closely resemble peritoneal metastases and create uncertainty, particularly when they are encountered unexpectedly during surgery. A lack of awareness may lead to overly aggressive surgical decisions or delays in definitive management.

**Case presentation:**

We describe a patient in whom abdominal splenosis was incidentally identified during the repair of an incisional hernia associated with a chronic discharging sinus. During exploration, numerous reddish-brown nodules were noted over the omentum and peritoneal surfaces, raising concern for peritoneal carcinomatosis, especially given the patient’s family history of colorectal cancer. A few representative nodules were excised and sent for histopathological examination. Intraoperative frozen section analysis was not performed, and therefore, the decision to proceed was based on gross intraoperative findings. In the absence of frozen-section confirmation, the decision to proceed with mesh repair was made based on the benign intraoperative appearance of the nodules (well-circumscribed, non-infiltrative, no ascites), and a clean operative field without evidence of infection. Considering these findings, the surgical team proceeded with definitive mesh repair of the hernia during the same operation. Histopathology later confirmed the presence of splenic tissue consistent with splenosis. The patient had an uneventful postoperative recovery.

**Conclusion:**

This case demonstrates that intraoperative recognition of splenosis, supported by careful assessment and limited biopsy, allows safe continuation of planned surgery without unnecessary delay or overtreatment. Awareness of this entity is essential to guide appropriate intraoperative decision-making and avoid misinterpretation as metastatic disease.

## Introduction

The unexpected identification of multiple peritoneal nodules during surgery often raises concern for metastatic disease and may influence intraoperative management [1, 2]. Nevertheless, not all such lesions are malignant. Abdominal splenosis is a benign condition resulting from autotransplantation of splenic tissue following splenic trauma or splenectomy, and it can closely resemble peritoneal metastasis, leading to diagnostic uncertainty [1]. A lack of awareness and failure to recognize splenosis intraoperatively may lead to unnecessary oncologic resection, abandonment of planned surgery, or additional procedures, thereby increasing patient morbidity.

Splenosis develops when dispersed splenic fragments establish a vascular supply from adjacent tissues and persist as functioning ectopic splenic tissue [1]. Although most commonly found within the peritoneal cavity, ectopic implants have also been described in the liver, pelvis, thorax, and subcutaneous tissue [1, 3]. Because patients are usually asymptomatic, the condition is frequently detected incidentally during imaging or surgery [2]. The principal challenge lies in its radiologic resemblance to malignant disease. Multiple nodular lesions observed by imaging may simulate peritoneal carcinomatosis, lymphoma, or metastatic deposits, creating a diagnostic dilemma, especially when these lesions are encountered during surgery for unrelated pathology [2, 4–6].

We present a case of abdominal splenosis incidentally identified during surgical repair of an incisional hernia associated with a chronic discharging sinus. The unexpected intraoperative finding of multiple nodules raised suspicion of metastatic disease and posed a significant intraoperative diagnostic dilemma with important implications for surgical decision-making and patient safety.

This report highlights the importance of cautious intraoperative assessment and limited biopsy to avoid overtreatment and safely complete definitive surgical management.

## Case presentation

A 52-year-old male from the state of Tamil Nadu in southern India presented to the surgical outpatient department with complaints of swelling over a previous midline abdominal scar. The swelling gradually increased in size over time and was associated with persistent mucopurulent discharge from a sinus located within a hypertrophic scar. The patient had undergone splenectomy six years earlier following blunt abdominal trauma. There was no significant past medical illness. The patient’s family history was remarkable, with a history of colorectal carcinoma in a first-degree relative and no known hereditary disorders.

On physical examination, reducible swelling measuring approximately 8 × 6 cm was noted below the midline scar. The swelling increased upon coughing and was clinically suggestive of an incisional hernia. A hypertrophic scar with a small discharging sinus was present at the same site. No signs of bowel obstruction or strangulation were noted. Contrast-enhanced CT imaging of the abdomen revealed a 4.7 × 4.0 × 1.3 cm defect in the posterior sheath of the left rectus abdominis muscle with herniation of omental fat and small bowel loops. In addition, multiple small soft-tissue nodules measuring approximately 1–1.5 cm were observed along the peritoneal surfaces and omental folds.

The presence of these nodules poses a diagnostic challenge, as their appearance on imaging raised suspicion for possible metastatic deposits. The differential diagnoses considered included peritoneal metastasis, peritoneal tuberculosis, lymphoma, and splenosis.

**Fig. 1:**
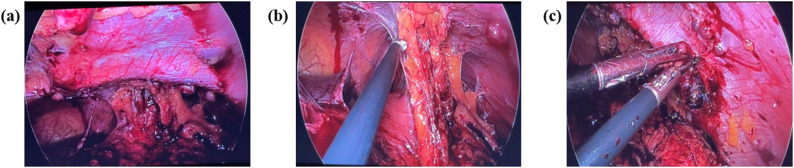
Diagnostic laparoscopy showing (**a**) omental adhesion to the peritoneum with a scattered nodular appearance of splenosis over the peritoneal layer of the abdomen. (**b**) Adhesiolysis was performed during diagnostic laparoscopy, and the contents were reduced. (**c**) Splenosis nodules were excised during diagnostic laparoscopy and sent for HPE

Given the diagnostic uncertainty, diagnostic laparoscopy was performed. Laparoscopic examination (Fig. 1a–c) revealed multiple reddish-brown nodules scattered over the peritoneal surfaces and omentum. Adhesions between the omentum and the anterior abdominal wall were also noted and carefully released. A few representative nodules were excised and sent for histopathological examination. The presence of multiple nodules, along with a family history of colorectal carcinoma, increased suspicion for metastatic disease.

**Fig. 2:**
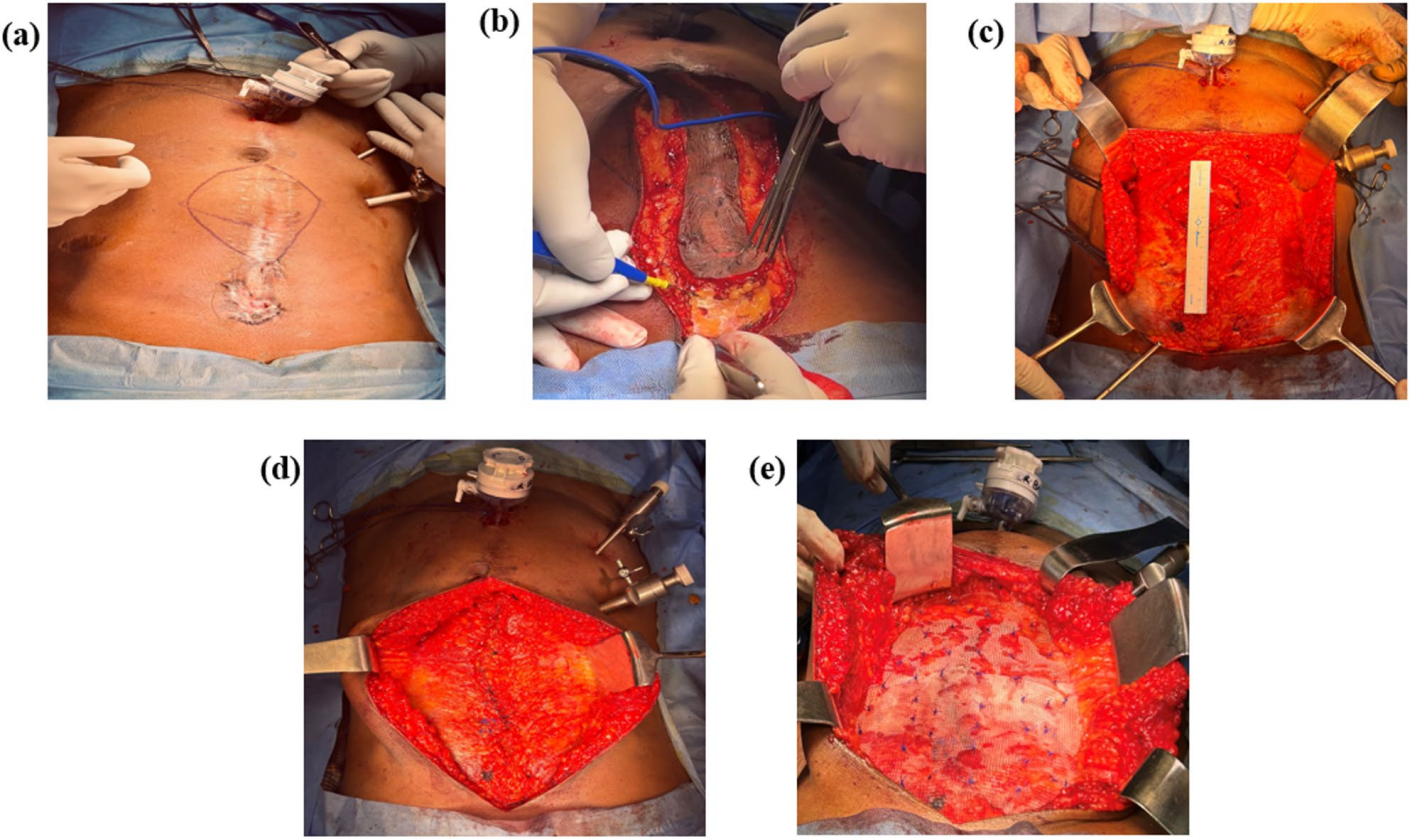
(**a**) An incision was made over the hypertrophic scar with the discharge sinus, (**b**) the hypertrophic scar was completely excised along with the discharge sinus, (**c**) the incisional hernia defect was identified, (**d**) the hernial defect was closed with 2–0 Ethilon, and (**e**) two 15 × 15 cm meshes were placed and fixed with 2–0 Prolene after closure of the hernial defect

Following laparoscopic assessment, an elliptical incision was made to excise the hypertrophic scar along the sinus tract. The incisional hernia defect was identified and repaired via 2–0 Ethilon sutures. Intraoperatively, the nodules appeared well circumscribed and non-infiltrative, with no evidence of ascites or peritoneal deposits suggestive of advanced malignancy. After careful inspection and excision of representative nodules for histopathological examination, the surgical field was assessed for contamination and was found to be clean. In view of the benign intraoperative appearance and absence of gross contamination, a decision was made to proceed with definitive mesh repair. Two 15 × 15 cm mesh prostheses were placed and secured with 2–0 Prolene sutures (Fig. 2). This approach allows safe completion of hernia repair while avoiding the need for staged surgery.

Histopathological examination of the excised nodules revealed splenic tissue composed of fibrocollagenous and adipose tissue with proliferating capillaries and areas of hemorrhage, confirming the diagnosis of abdominal splenosis. The postoperative recovery was uneventful. The drain was removed on postoperative day four. The sutures were removed on postoperative day fourteen, and the wounds healed satisfactorily. At the three-month follow-up, the patient remained asymptomatic, with no evidence of recurrence or postoperative complications.

## Discussion

Abdominal splenosis is a benign condition resulting from the heterotopic implantation of splenic tissue following splenic trauma or splenectomy [1]. The reported incidence ranges from 16% to 65% among patients who have experienced splenic injury [5]. Unlike accessory spleens, splenosis nodules do not possess a true capsule or hilum and obtain their blood supply from surrounding tissues rather than the splenic artery [7]. A significant diagnostic challenge arises because splenosis lesions can closely resemble malignant disease on imaging. These nodules may appear similar to peritoneal metastases, lymphoma, or hepatic tumors [2, 6].

In the present case, CT imaging revealed multiple peritoneal nodules that initially raised suspicion of metastatic disease. Diagnostic laparoscopy allows direct visualization of the lesions and enables biopsy for histopathological confirmation. Several studies have described abdominal splenosis presenting many years after splenic trauma or splenectomy. Vernuccio et al. reported that splenosis may mimic other intra-abdominal pathologies on imaging, often leading to diagnostic uncertainty [1, 3]. Similarly, Chapagain et al. described cases where multiple intra-abdominal nodules were initially suspected to represent malignant lesions before splenosis was confirmed [2]. Liu et al. also reported that abdominal splenosis presented findings that resembled those of hepatic or intestinal tumors [6]. Previous reports in Patient Safety in Surgery have highlighted that careful intraoperative reassessment and avoidance of irreversible decisions are key strategies to reduce preventable surgical harm [9].

The presence of multiple peritoneal nodules initially suggested metastatic disease, but postoperative histopathological examination confirmed splenosis. Adherence to surgical safety principles, including careful intraoperative reassessment and avoidance of irreversible decisions without diagnostic confirmation, aligns with WHO recommendations to minimize preventable surgical harm. Recognizing this entity intraoperatively is essential to avoid inappropriate surgical decisions, such as unnecessary biopsy, abandonment of planned procedures, or delay in definitive management. Definitive oncologic resection would not be undertaken without prior diagnosis, staging, and multidisciplinary planning.

A key intraoperative concern in this case was the placement of prosthetic mesh in the presence of undiagnosed peritoneal nodules. If these lesions had represented metastatic disease, mesh placement could potentially complicate future oncological surgery due to fibrosis, adhesions, and difficulty in re-entry. In this context, the decision to proceed was made cautiously, based on the benign intraoperative characteristics of the nodules and absence of disseminated malignant features. This highlights the importance of careful intraoperative judgment when balancing the risks of delaying definitive hernia repair against the potential implications for future oncologic management.

A limitation of this report is that preoperative nuclear scintigraphy, which can noninvasively identify ectopic splenic tissue, was not performed [8]. Such imaging may have helped establish the diagnosis preoperatively and reduced intraoperative diagnostic uncertainty. However, histopathological examination provides definitive confirmation, and intraoperative decision-making allows safe completion of the procedure.

From a patient safety perspective, misinterpretation of these nodules as peritoneal metastases could have led to unnecessary extensive oncologic resection or abandonment of definitive hernia repair [2, 6]. Intraoperative recognition of the benign appearance and performance of limited biopsy enabled confirmation of the diagnosis while avoiding overtreatment, consistent with previously described patient safety strategies [9]. This approach allows safe mesh placement and completion of hernia repair during the same procedure, thereby preventing additional surgery, reducing patient morbidity, and optimizing surgical outcomes.

## Conclusion

Abdominal splenosis is a benign condition that may closely mimic peritoneal metastatic disease. This case highlights the diagnostic challenge when splenosis is encountered during incisional hernia repair. Diagnostic laparoscopy with biopsy allows definitive diagnosis and enables safe simultaneous surgical management. Awareness of this entity can prevent misdiagnosis and unnecessary oncologic procedures.

## Data Availability

No datasets were generated or analysed during the current study.
